# Diagnostic performance of clinical properties and conventional magnetic resonance imaging for determining the *IDH1* mutation status in glioblastoma: a retrospective study

**DOI:** 10.7717/peerj.7154

**Published:** 2019-06-21

**Authors:** Qun Wang, Jiashu Zhang, Fangye Li, Xinghua Xu, Bainan Xu

**Affiliations:** Department of Neurosurgery, Chinese PLA General Hospital, Beijing, Beijing, China

**Keywords:** Isocitrate dehydrogenase 1 (*IDH1*), Age, Tumor size, Glioblastoma, Magnetic resonance imaging

## Abstract

**Background:**

Glioblastoma (GBM), the most malignant form of gliomas, is a relatively common primary brain tumor in adults. Preoperative identification of isocitrate dehydrogenase 1 (*IDH1*) mutations in GBM is of critical prognostic importance. The aim of the present study was to explore the feasibility and diagnostic performance of basic patient information combined with conventional magnetic resonance imaging (MRI) findings for determination of the *IDH1* status (mutant vs wild type) in patients with GBM.

**Methods:**

From January 1, 2016 to December 31, 2017, a consecutive series of 50 patients with GBM was retrospectively collected. The patients were divided into two group according to their *IDH1* mutation status. Basic information and MRI features were analyzed for the establishment of a diagnostic prediction model using logistic regression. A receiver operating characteristic curve was used to evaluate the diagnostic performance.

**Results:**

Patients with *IDH1-*mutant tumors were younger than those with *IDH1*-wild type tumors, and exhibited a larger tumor volume. The diagnostic predictive model established by combining age and the tumor size exhibited a sensitivity and specificity of 70% and 93%, respectively. The area under the curve was 0.88, which indicated high diagnostic performance.

**Conclusion:**

Patient age and tumor volume can be used as indicators of *IDH1* mutation status in patients with GBM, with high diagnostic performance for simple evaluations in clinical practice. The combined use of these two indicators can further enhance the diagnostic specificity.

## Introduction

Glioblastoma (GBM) is the most common and malignant form of gliomas and is associated with an extremely poor prognosis. The median overall survival time is 15 months after maximal tumor resection and chemoradiotherapy (Dolecek et al., 2012). However, because of individual differences among tumors and patients, some patients survive for a few months while others survive for several years (Gerber et al., 2014). The isocitrate dehydrogenase 1 (*IDH1*) mutation status is a powerful, independent predictor of glioma-related survival. Patients with *IDH1*-mutant astrocytomas can expect a better prognosis than can patients with *IDH1*-wild type astrocytomas of different grades. In particular, patients with *IDH1*-wild type GBM reportedly exhibited a predicted survival rate that is 25% of that exhibited by those with *IDH1*-mutant tumors (Ahmadipour et al., 2019). Parsons et al. (2008) reported that patients with *IDH*-mutant GBM had an average survival time of 3.8 years, while the average survival time of *IDH1*-wild type patients was 1.1 years. These reports indicated that the *IDH1* mutation status in patents with GBM was significantly correlated with the prognosis, and that preoperative identification of *IDH1* mutations in GBM is of critical prognostic importance.

In the diagnosis of gliomas, immunohistochemistry and genomic sequencing are considered “gold standard” pathological and molecular methods for detecting *IDH1* mutations (Kickingereder et al., 2015). However, these methods require histopathological specimens that must be collected using invasive methods. In addition, biopsies may provide incorrect results because of intratumoral heterogeneity, such that it becomes impossible to reliably assess the overall tumor status. Early and correct diagnosis is critical to clinical treatment and prognosis; the most readily-available tumor features are often revealed by a careful assessment of the patient’s clinical presentation and magnetic resonance (MR) images.

The present study explored whether combination of basic patient information and conventional magnetic resonance imaging (MRI) finding can be used to determine the *IDH1* status (mutant vs wild type) in GBM patients. This information was also used to establish a predictive diagnostic model for the assessment of *IDH1*-mutant subtypes of GBM, in order to promote more accurate diagnosis and treatment.

## Materials and Methods

### Participants and data collection

A total of 221 consecutive patients were screened in this retrospective study; all were admitted to our department for treatment and were pathologically diagnosed with gliomas between January 1, 2016 and December 31, 2017. The Medical Ethic Committee of Chinese PLA General Hospital had approved this study. The study inclusion criteria were as follows: (1) patients who were admitted to the hospital with complete clinical information, including age, gender, and imaging data; (2) patients who underwent routine preoperative T1- and T2-weighted imaging, as well as contrast-enhanced T1(T1+C)—and T2-fluid attenuated inversion recovery (FLAIR) imaging; (3) patients who had a pathological diagnosis of GBM after surgical resection, with a clear molecular pathological classification and grading according to the 2016 WHO standard; (4) and patients who had a first-time diagnosis without a history of surgery, radiotherapy, or chemotherapy. The study exclusion criteria were as follows: (1) patients who had incomplete or low quality preoperative MRI data before surgery; and (2) patients whose images were poorly registered during image processing.

Of the 221 patients assessed for study inclusion, 59 were pathologically diagnosed with GBM, nine of these were further excluded in accordance with the above exclusion criteria; seven because of incomplete and/or low quality MR images, one because of confusing immunohistochemical findings, and one because of poor image registration during image processing. Eventually 50 patients including 35 men and 15 women (average age, 48.8 years) were enrolled. According to the presence or absence of the *IDH1* mutation, the patients were divided into *IDH1*-mutant and *IDH1*-wild type groups for further analyses.

### Image acquisition

Anatomical imaging sequences included T1-weighted, T2-weighted, T2-FLAIR, and T1+C images, all of which were acquired using a 1.5-T high-field-strength superconducting magnet in an iMRI system diagnostic room (Espree, Siemens, Germany) (Chen et al., 2012). Examination parameters for each were as follows: for T1, repetition time/echo time (TR/TE) 2,020/4.38 ms; layer thickness, one mm; field of view (FOV), 250 × 250 mm; sequence duration, 5 min 30 s; image resolution, 1 × 1 × 1 mm; for T2, TR/TE, 4,000/97 ms; layer thickness, three mm; FOV, 230 × 230 mm; sequence duration, 2 min; image resolution, 0.7 × 0.7 × 4 mm; and for T2-FLAIR, TR/TE, 9,000/84 ms; layer thickness, five mm; sequence duration, 4 min; image resolution, 0.45 × 0.45 × 5.5; For T1 plus; C imaging, the parameters were identical to those used for T1, with gadopentetate dimeglumine as the contrast agent.

### Imaging registration, tumor segmentation, and volume calculation

Patient MRI data were imported into 3DSlicer software (www.slicer.org) in the DICOM format. Sequence resampling was performed using the Resample Scalar Volume module in order to ensure equivalent layer thickness and voxel numbers by referencing the T2-FLAIR sequence (the FLAIR sequence displays tumors well and is thus helpful for tumor segmentation) and setting each voxel to a fixed volume of 1 × 1 × 5.235 mm. The BRAINS module of the 3DSlicer software was used for linear registration of rigid bodies between sequences. Further details regarding the procedure can be found on the following website: https://www.slicer.org/wiki/Documentation/Nightly/Registration/RegistrationLibrary.

**Figure 1:**
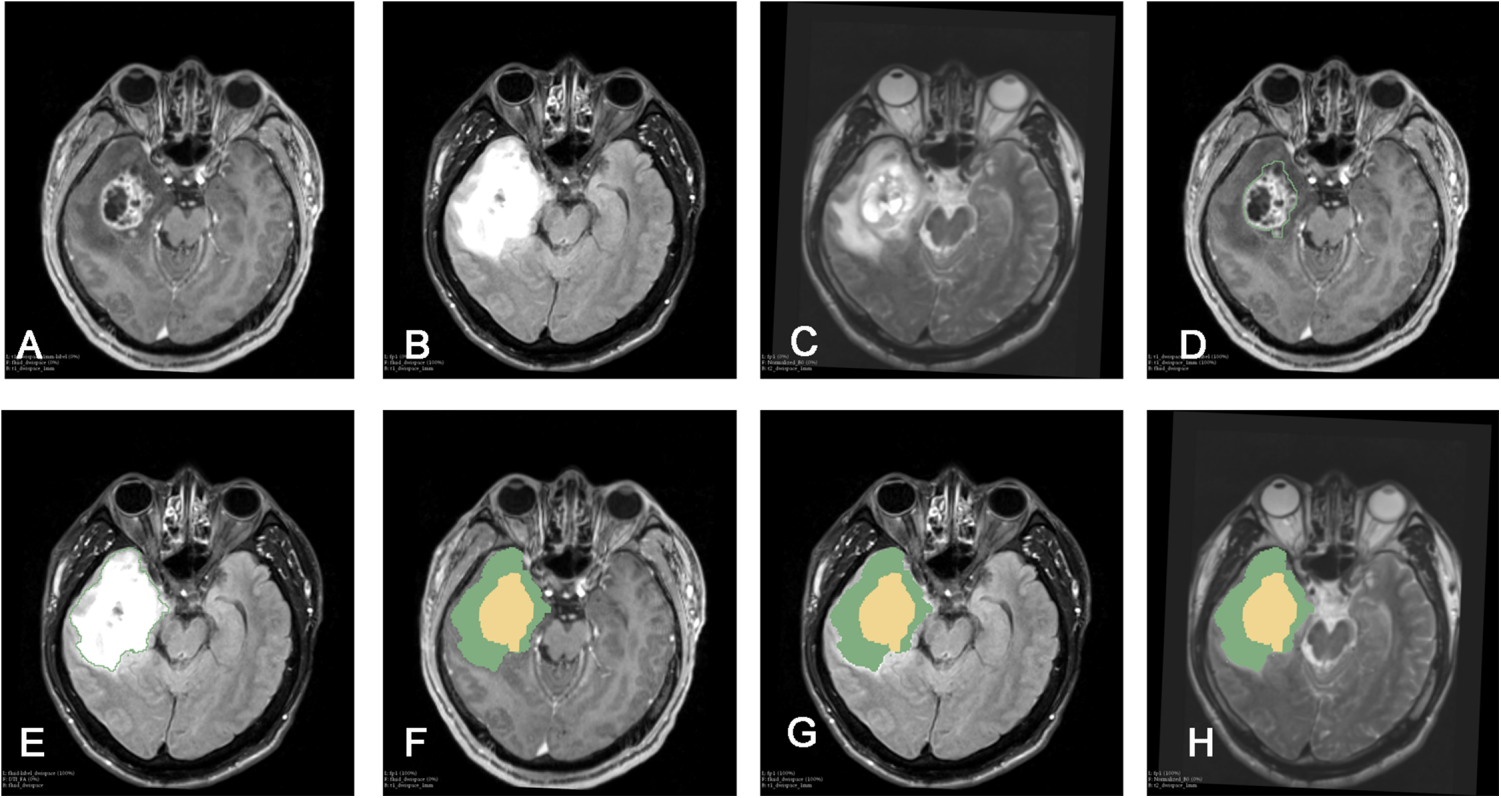
Procedure of tumor segmentation of Case. Case: 43 years old, male, headache for more than half a month, MRI showed right temporal lobe lesions, after surgical resection, pathological examination reported for WHO IV glioblastoma, IDH wild type. As shown in the figure, patient MRI T1enhancement, T2 and T2-FLAIR were shown, after importing into 3D Slicer software, Editor module was used to segment the tumor: (A) T1+C shows tumor enhancement; (B) T2-FLAIR sequence; (C) T2 shows the tumor area; (D) Segmentation of the tumor-enhanced part according to the T1 enhancement sequence in the Slicer software is shown as Label-1; (E) Segmentation of the whole tumor according to the T2-FLAIR is shown as Label-2; (F) T1+C shows Label-3. In the Add Scalar Volume module of Slicer, Label-1 (enhanced part) and Label-2 (tumor whole) are superimposed to obtain the enhanced part (yellow) and the tumor edema part, respectively. (Green) Label-3; (G) T2-FLAIR shows the enhanced part (yellow) and tumor edema (green) after segmentation, respectively; (H) T2 image shows the enhanced part (yellow) and tumor edema, respectively Part (green).

Whole tumors were segmented in the Editor module according to the FLAIR images. Segmented tumor images were referred to as Label-1, representative of the whole tumor. Contrast-enhanced regions of the tumor were segmented using the T1+C sequence and referred to as Label-2. Using the software’s Add Scalar Volume module, the whole tumor (Label-1) was superimposed onto the tumor-core region (Label-2) to obtain a new image. This was referred to as Label-3, and showed both the tumor-edema region and the tumor-core region. The volumes of the tumor-edema region and the tumor-core regions were separately calculated using the data statistic module Label Statistic in 3DSlicer software. Figure 1 depicts this procedure.

### Imaging information evaluation

Basic patient data, including age, sex, and the pathological diagnosis, were collected. Two deputy chief physicians of neuroradiology, who were blinded to all patient’s pathological background recorded and analyzed the location and laterality of all lesions, and determined the presense of lesion enhancement and cystic necrosis. Basic patient information, imaging data, and tumor volume statistics were integrated. Disagreements or controversies regarding the imaging data were resolved though communication and consultation until a consensus was reached.

### Pathological examination

All pathological specimens were obtained by surgical resection and diagnosed by neuropathologists in the Department of Pathology. Diagnostic criteria were based on the 2016 World Health Organization classification of tumors of the central nervous system (Louis et al., 2016). The pathological specimens were subjected to routine staining and immunohistochemical analyses, and the *IDH1* mutation status was confirmed by *IDH1* R132 antibody (internal clone H09; Dianova, Hamburg, Germany).

### Statistical methods

Data processing and analysis was performed using the EmpowerStats software (www.empowerstats.com, X&Y Solutions, Inc., Boston, MA, USA). Graphs were generated using GraphPad Prism 5 (Version 5.01, GraphPad, La Jolla, CA, USA). Differences between the *IDH*-mutant and *IDH*-wild type groups were statistically evaluated, with continuous variables subjected to the Kruskal–Wallis rank sum test and categorical variables subjected to the chi-square test. If a count variable had a theoretical value of <10, Fisher’s exact probability test was used. For extremely complex analyses, such as the analysis of predictive equations in the diagnostic model, the advanced diagnostic module of the EmpowerStats software was employed. In cases of statistical differences, receiver operating characteristic (ROC) curves were generated, and the sensitivity, specificity, positive predictive value, and negative predictive value were calculated. All tests were two-sided hypothesis tests, with the test level set to α = 0.05.

## Results

### Participant demographics

Demographic information and characteristics for the *IDH1*-mutant and *IDH1*-wild type groups were separately recorded and analyzed (Table 1). There was a significant difference in age, the tumor location, and the overall tumor volume between the two groups. There were no significant differences between the two groups with regard to sex, tumor laterality, the presence of lesion enhancement, the presence of cystic change, or the tumor-edema and tumor-core volume. Moreover, there was a significant difference in terms of tumor distribution in the cerebral lobes: *IDH1*-mutant tumors were mainly located in the temporal (50%) and frontal (40%) lobes, whereas *IDH1*-wild type tumors were most often located in the temporal lobe (47.5%).

**Table 1:** The detailed characteristics of the patients.

Characteristics	*IDH-1* status		*P*-value
	Wild type	Mutant type	
Cases number	40	10	
Age (years)	52.3 ± 11.6	34.8 ± 10.8	<0.001
Gender			0.44
Female	13 (32.50%)	2 (20.00%)	
Male	27 (67.50%)	8 (80.00%)	
Tumor side			0.827
Bilateral	2 (5.00%)	1 (10.00%)	
Left	18 (45.00%)	4 (40.00%)	
Right	20 (50.00%)	5 (50.00%)	
Tumor location			<0.001
Frontal	11 (27.50%)	4 (40.00%)	
Temporal	19 (47.50%)	5 (50.00%)	
Parietal	5 (12.50%)	0 (0.00%)	
Occipital	1 (2.50%)	0 (0.00%)	
Insula	3 (7.50%)	1 (10.00%)	
other	1 (2.50%)	0 (0.00%)	
Contrast enhancement			0.118
Yes	2 (5.00%)	2 (20.00%)	
No	38 (95.00%)	8 (80.00%)	
Necrosis			0.177
Yes	5 (12.50%)	3 (30.00%)	
No	35 (87.50%)	7 (70.00%)	
Tumor core volume (cm^3^)	9.74 ± 6.40	13.67 ± 11.30	0.396
Edema volume (cm^3^)	15.37 ± 8.51	18.83 ± 12.63	0.303
Total volume (cm^3^)	25.11 ± 10.44	32.51 ± 10.63	0.042

### Relationship between patient age and *IDH1* mutation status

**Figure 2:**
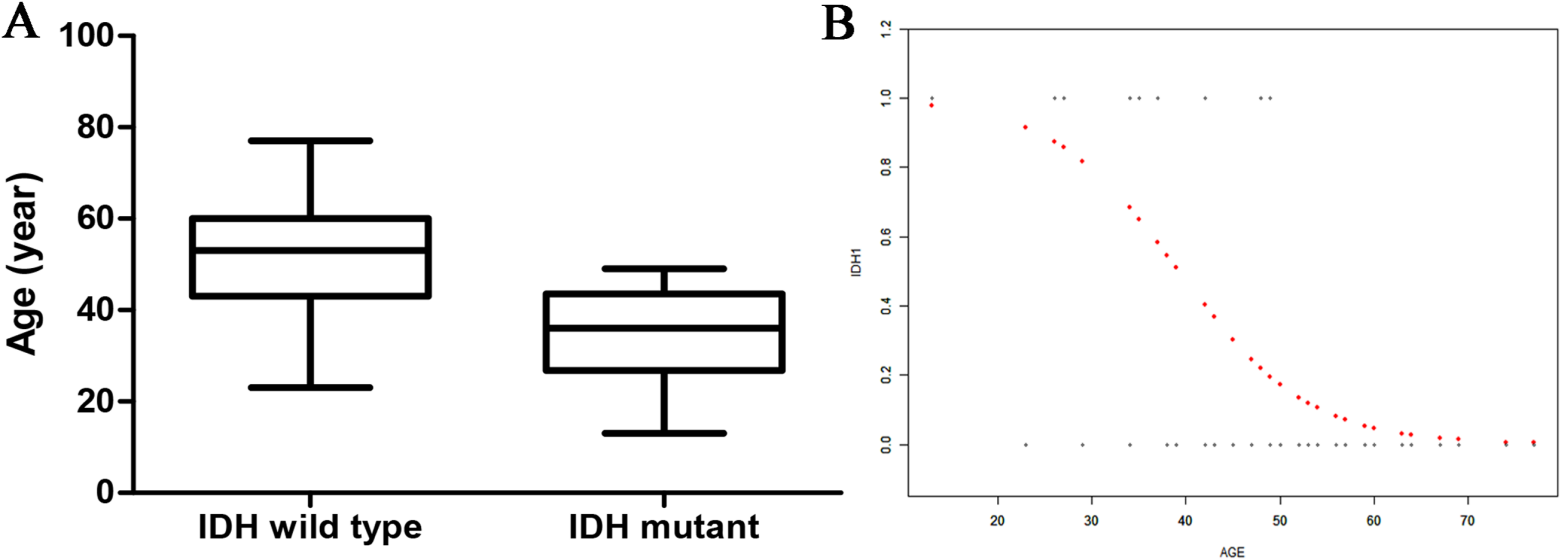
The relationship of age and IDH status. (A) Boxplot of tumor size (separated by IDH status); (B) Smooth curve fitting plot of IDH status and age.

There was a statistical difference in age between the two groups: the *IDH1*-mutant patients were younger than the *IDH1*-wild type patients (34.8 ± 10.8 vs 52.3 ± 11.6 years) (Fig. 2A). Curve fitting analysis of the patient age compared with the *IDH1* mutation status revealed that the probability of an *IDH1*-wild type status in a GBM patient gradually increased with an increase in age (Fig. 2B).

### Relationship between tumor volume and *IDH1* mutation status

**Figure 3:**
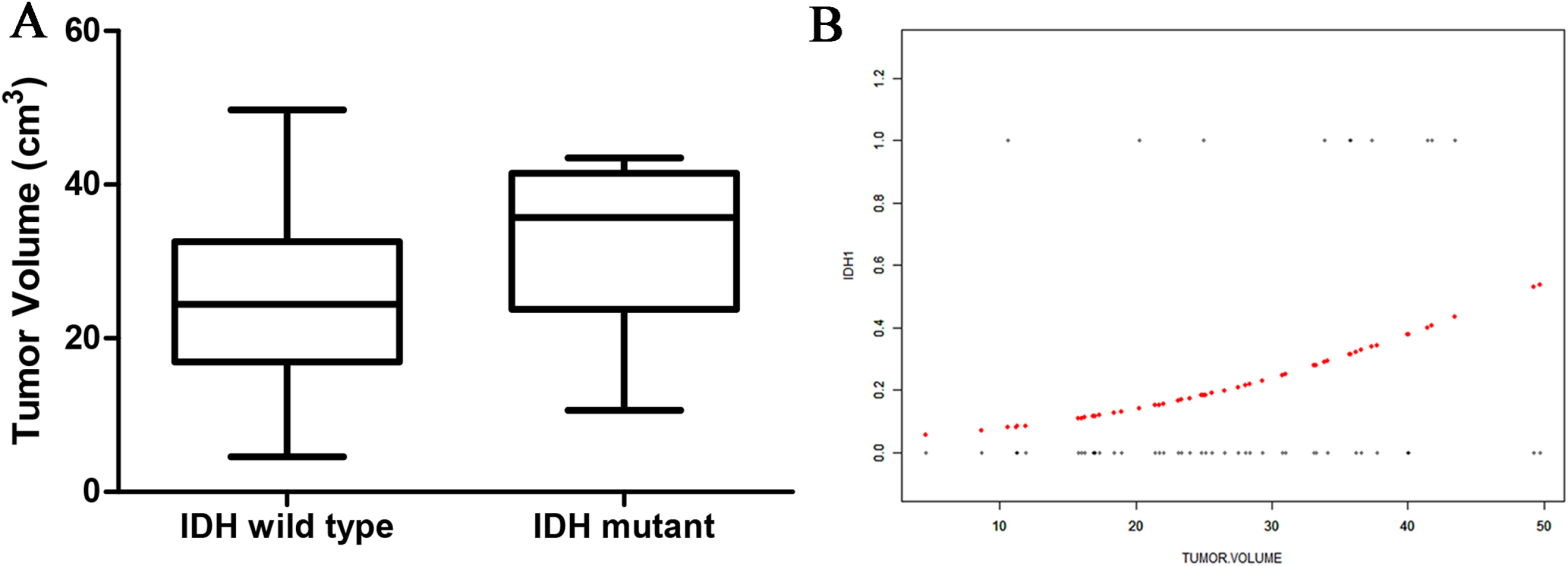
The relationship of tumor size and IDH status. (A) Boxplot of tumor size (separated by IDH status); (B) Smooth curve fitting plot of IDH status and tumor size.

There was no significant difference in the tumor-edema and tumor-core volumes between the two genotype groups. However, there was a significant difference in the overall tumor volume between the two groups: *IDH1*-mutant patients exhibited larger overall tumor volumes than did *IDH1*-wild type patients (32.51 ± 10.63 vs 25.11 ± 10.44 cm^3^; Fig. 3A). Curve fitting analysis of the tumor size compared with the *IDH1* mutation status revealed that the probability of an *IDH1*-mutant status in a GBM patient gradually increased with an increase in the tumor volume (Fig. 3B).

### ROC curve analyses

**Figure 4:**
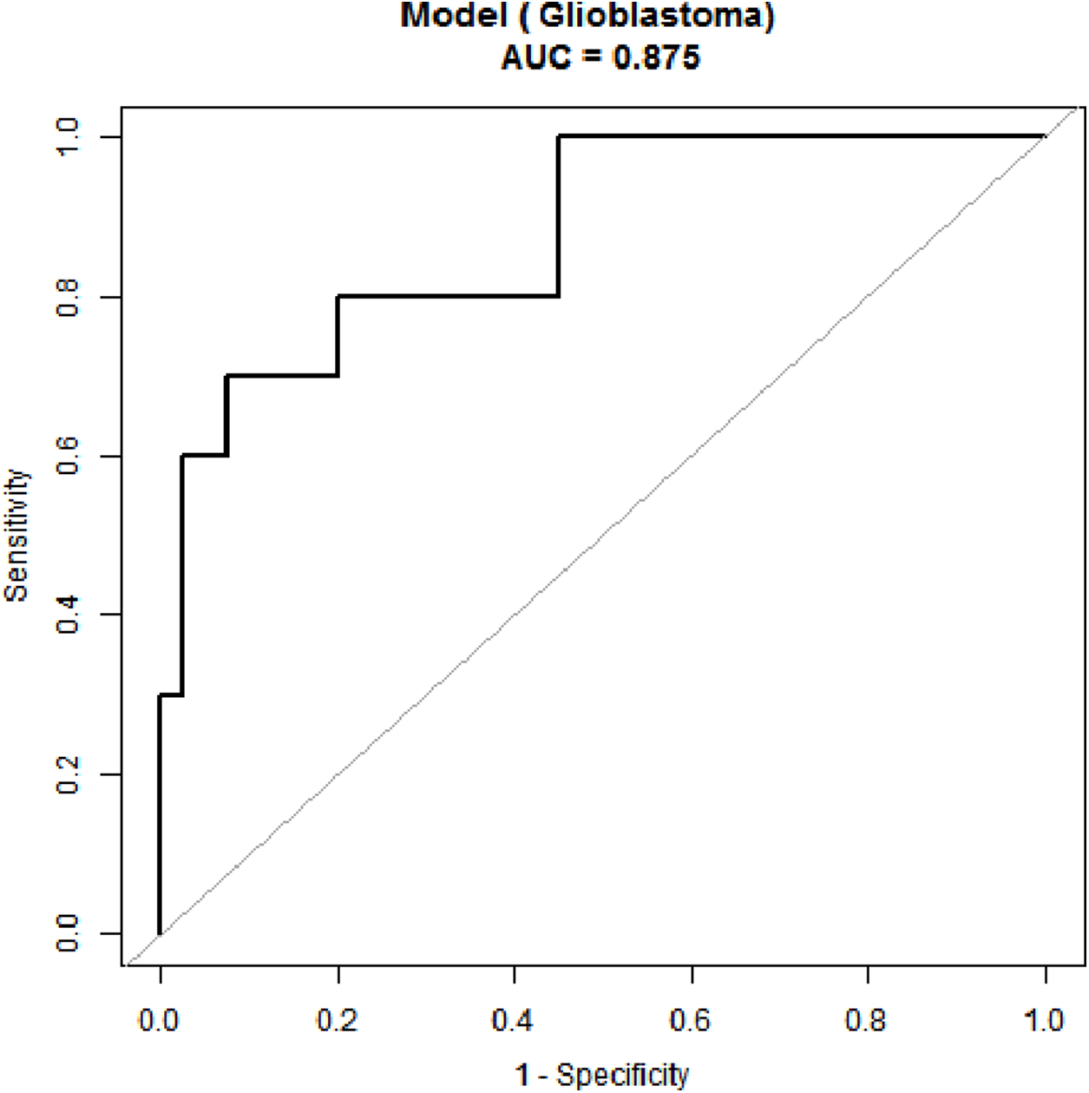
Receiver operating characteristic (ROC) curve for IDH status prediction combining with age and tumor size.

Receiver operating characteristic curve analyses were performed for age and the tumor volume, the two parameters that significantly differed between the two groups (Fig. 4). When the age threshold was set to 42.5 years, the sensitivity and specificity of our model were 80% and 82%, respectively, and the AUC was 0.87. When the tumor volume threshold was set to 33.6 cm^3^, the sensitivity and specificity were 70% and 80%, respectively, and the AUC was 0.80 (Table 2). The following logistic regression model was used to establish a diagnostic prediction model that incorporated both age and the tumor volume for joint diagnosis: logit(*IDH1*) = 2.16674 − 0.13007 × Age + 0.07703 × Tumor volume. When the threshold was set to −0.89, the sensitivity and specificity were 70% and 93%, respectively, and the AUC was 0.88 (Table 2).

**Table 2:** The diagnostic performance of the different parameters.

Test	AUC	Threshold	Spe	Sen	Accuracy	PLR	NLR	PPV	NPV	DOR
Age	0.87 (0.74, 0.96)	42.5	0.82	0.8	0.82	4.57	0.24	0.53	0.94	18.86
Tumor volume	0.70 (0.52, 0.88)	33.55	0.8	0.7	0.78	3.5	0.38	0.47	0.91	9.33
Model	0.88 (0.75, 1)	−0.89	0.93	0.70	0.88	9.33	0.32	0.70	0.93	28.78

## Discussion

In the present study, we compared clinical data and conventional MRI data between *IDH1*-mutant and *IDH1*-wild type GBM patients and found significant differences in age, the tumor location, and the tumor size between the two groups. Moreover, we combined clinical data with conventional imaging features to facilitate determination of *IDH1* mutation status. Our results provided a simple method for accurate, early (preoperative), and noninvasive evaluation of *IDH1* mutation status in GBM patients.

Noninvasive preoperative assessment of the genetic subtypes of the *IDH1* mutation in glioma patients is likely to enable more effective selection of clinical treatment options. Beiko et al. (2014) reported that patients with *IDH1*-mutant gliomas had better prognoses after complete tumor resection, while the prognosis of patients with *IDH1*-wild type tumors was less correlated with the degree of tumor resection, suggesting that *IDH1*-mutant patients might benefit more from aggressive tumor surgery. Furthermore, the *IDH1* mutation status affected both the prognosis of the glioma and the patient’s response to radiotherapy and chemotherapy. Reitman & Yan (2010) reported that patients with *IDH1*-mutant GBM were more sensitive to temozolomide. However, Jafari-Khouzani et al. (2016) reported that *IDH1*-mutated gliomas exhibited better radiosensitivity and experienced greater benefits from postoperative chemotherapy; these finding were corroborated by Okita et al. (2012).

The mean age of *IDH1*-mutant GBM patients in the present study was significantly lower than that of *IDH1*-wild type GBM patients. Curve fitting analysis revealed that the probability of an *IDH1* mutation decreased with an increase in age. This finding was consistent with that in a prior study of 276 cases of primary GBM, which revealed that *IDH1*-mutant GBM patients were often younger than their wild-type comparators (Popov et al., 2013). Leibetseder et al. (2013) also reported that *IDH1* mutations were more likely in younger patients, thus indicating that age is at least particularly associated with the *IDH* mutation status in GBM.

The mechanisms underlying the ralationship between age and the GBM status are poorly understood. One study found that telomerase activation in GBM tissues occurred only in younger GBM patients, mostly with *IDH1* mutations. This implies that telomerase may play a role in the relationship between the mutation status and age (Lötsch et al., 2013). Another study revealed that the preferential of the *IDH1*-mutant GBM in young patients may be related to the influence of mutations in the gene encoding variant histone H3.3 on GBMs in children (Popov et al., 2013). Thus far, the effects of age on the *IDH1* mutation status remain unclear, and there is an urgent need for in-depth research on the molecular pathology underlying this relationship.

There was a significant difference in the overall tumor volume between the *IDH1*-mutant and *IDH1*-wild type groups in the present study, although no significant differences in the tumor-edema and tumor-core volumes were observed. Thus, *IDH1*-mutant GBM patients featured a larger tumor volume, consistent with the finding in a large cohort study of GBM patients (Lai et al., 2011). Using an open database of brain tumor segmentation, Eichinger et al. (2017) found that, among grade II and III gliomas, *IDH1*-mutant tumors were larger than *IDH1*-wild type tumors. This may be related to the location of *IDH1*-mutant tumors, which mainly occur in the frontal lobe; thus, these patients do not exhibit clinical symptoms until the tumor has grown much larger. In addition, *IDH*-mutant tumors grow at a slower rate than do *IDH1*-wild type tumors, thereby allowing for brain compensation and plasticity to occur, which further delaying diagnosis.

When diagnosing the *IDH1* mutation status in GBM patients on the basis of age alone, we found a sensitivity and specificity of 80% and 82%, respectively. These values were 70% and 80%, respectively, when the tumor volume was used alone. However, combination of age and tumor volume revealed that the specificity with which *IDH1* mutations in GBM may be identified increased to 93%. These results indicate the power of a combined predictive model.

In summary, we found that the IDH1 mutation status may be determined using a combination of age and tumor volume in patients who are diagnosed with GBM via traditional medical imaging methods. This is a noninvasive and efficient method for clinical evaluation. However, the study had some limitations. Although the combination model exhibited a moderate diagnostic performance, the sensitivity decreased when the two indicators were combined. Moreover, *IDH1* mutations are rare in GBM patients; therefore, only 10 *IDH*-mutant GBM patients were included in the present study. This small sample size may have introduced sampling bias and consequently interfered with our results. Another limitation was the retrospective design. The IDH1 mutation status was confirmed by immunohistochemistry without genetic testing; this may have resulted in our overlooking some *IDH1*-positive patients. In the future, large-sample prospective studies are needed to determine diagnostic age thresholds for the broader Chinese population for more accurate determination of the *IDH1* genotype and prognostic status of GBM patients. Moreover, the associations between the age- and tumor volume-based GBM pathophysiological characteristics of GBM and the pathoetiological mechanisms underlying the *IDH1* mutation status need further investigation.

## Conclusions

In the present study, we found that age and the tumor volume may be used to indicate *IDH1* mutation status in GBM patients with a high degree of diagnostic performance. The combined use of these two indicators may further enhance the diagnostic specificity, thus facilitating noninvasive, efficient, and accurate clinical evaluation.

## Supplemental Information

10.7717/peerj.7154/supp-1 Supplemental Information 1 The raw data (clinical information and conventional MRI features) of the 50 GBM patients.

## Additional Information and Declarations

### Data Availability

The following information was supplied regarding data availability: The information of the raw data showing 221 glioma patients’ clinical and tumor features are available as a Supplemental File.
